# A randomized controlled trial of a team science intervention to enhance collaboration readiness and behavior among early career scholars in the Clinical and Translational Science Award network

**DOI:** 10.1017/cts.2023.692

**Published:** 2023-12-14

**Authors:** Larry W. Hawk, Timothy F. Murphy, Katherine E. Hartmann, Andy Burnett, Eugene Maguin

**Affiliations:** 1 Department of Psychology, University at Buffalo, Buffalo, NY, USA; 2 Jacobs School of Medicine & Biomedical Sciences, University at Buffalo, Buffalo, NY, USA; 3 Clinical and Translational Science Institute, University at Buffalo, Buffalo, NY, USA; 4 Department of Obstetrics and Gynecology, Vanderbilt University, Nashville, TN, USA; 5 Department of Medicine, Vanderbilt University, Nashville, TN, USA; 6 Vanderbilt Institute for Clinical and Translational Research, Vanderbilt University, Nashville, TN, USA; 7 KnowInnovation, Inc., Seminole, FL, USA

**Keywords:** Team science, intervention, collaboration, early career scholars, training

## Abstract

**Introduction:**

Despite the central importance of cross-disciplinary collaboration in the Clinical and Translational Science Award (CTSA) network and the implementation of various programs designed to enhance collaboration, rigorous evidence for the efficacy of these approaches is lacking. We conducted a novel randomized controlled trial (RCT; ClinicalTrials.gov identifier: NCT05395286) of a promising approach to enhance collaboration readiness and behavior among 95 early career scholars from throughout the CTSA network.

**Methods:**

Participants were randomly assigned (within two cohorts) to participate in an Innovation Lab, a week-long immersive collaboration experience, or to a treatment-as-usual control group. Primary outcomes were change in metrics of self-reported collaboration readiness (through 12-month follow-up) and objective collaboration network size from bibliometrics (through 21 months); secondary outcomes included self-reported number of grants submitted and, among Innovation Lab participants only, reactions to the Lab experience (through 12 months).

**Results:**

Short-term reactions from Innovation Lab participants were quite positive, and controlled evidence for a beneficial impact of Innovation Labs over the control condition was observed in the self-reported number of grant proposals in the intent-to-treat sample. Primary measures of collaboration readiness were near ceiling in both groups, limiting the ability to detect enhancement. Collaboration network size increased over time to a comparable degree in both groups.

**Conclusions:**

The findings highlight the need for systematic intervention development research to identify efficacious strategies that can be implemented throughout the CTSA network to better support the goal of enhanced cross-disciplinary collaboration.

Cross-disciplinary collaborations, which generate more innovative, higher-impact science [1], are particularly important for clinical and translational research because the development, evaluation, and implementation of new interventions require contributions from multiple disciplines. Thus, the National Center for Advancing Translational Sciences (NCATS) emphasizes both cross-disciplinary and cross-institution collaboration within and across its Clinical and Translational Science Award (CTSA) hubs [2,3].

Effective initiation and maintenance of cross-disciplinary collaborations face many challenges [4,5]. The Science of Team Science (SciTS) field has produced helpful frameworks and described individual and team characteristics and practices to address these challenges [4–6]. Although preliminary evidence supports a range of team science interventions, rigorous evidence for the efficacy of these treatments is meager [6–9]. Studies of team science interventions are typically single-arm, pre- and post-observational studies with small sample sizes and are limited to self-reported outcomes without follow-up beyond the intervention. As Rolland and colleagues summarize in the introduction to a recent *Journal of Clinical and Translational Science* themed issue, the situation is far from ideal: “…the relative dearth of evidence-based interventions…can leave translational scientists to fend for themselves in establishing effective teams” [6] (p. 1).

In response to calls to strengthen the evidence base for team science interventions [4,10], this study conducted a randomized controlled trial (RCT) of a promising approach to enhance collaboration readiness and behavior. Two cohorts of scholars recruited across the CTSA network applied to attend 5-day residential *Innovation Labs*. Innovation Labs typically engage a group of 25-30 participants from a broad range of disciplines and training backgrounds in a facilitated journey through the creative problem-solving process to develop new transdisciplinary teams who rapidly develop and refine novel proposals to address a grand challenge in science [11]. The Innovation Lab structure and process was originally developed in 2003 by the United Kingdom’s Engineering and Physical Sciences Research Council, in partnership with KnowInnovation, a creativity research and facilitation organization that was also the industry partner on the present study. Since 2003, Innovation Labs (a.k.a. “sandpits” and “ideas labs” depending on the funding agency and parameters) have been hosted by NIH, NSF, and NASA on a wide range of problems (e.g., synthetic biology, origins of life, mobile health, cell behavior in cancer, cancer risk behavior) [11–17]. Innovation Labs are well received by participants, host organizations, and funding agencies and appear effective for fostering new transdisciplinary teams who generate innovative research that is well-funded [11].

Importantly, the participants in Innovation Labs have not typically previously collaborated with one another, and teams form and evolve organically, with some team members leaving to join other teams and other participants being added to address specific proposal needs as the week progresses. We harnessed the Innovation Labs framework as an opportunity for experiential learning in establishing new cross-disciplinary collaborations. Based on guidance from NCATS, our study focused on early career investigators, a critical part of the clinical and translational research workforce. Given their potentially limited scholar networks and practical experience developing collaborations, as well as their potential disadvantage in obtaining NIH funding [18], early career scholars may benefit substantially from a team science/collaboration intervention. Top-ranked applicants were randomly assigned to the Innovation Lab experimental group or to a treatment-as-usual (TAU) control group (i.e., the naturally occurring activities at their home institutions).

Major advances from this study are the randomized design and a stronger assessment frame. In contrast to the standard Innovation Lab, which focuses on the specific teams and proposals formed during the Lab, we targeted broader metrics of collaboration readiness and behavior. Following the logic model of Masse *et al*. [19], we focused on intermediate outcome markers. Specifically, we assessed self-reported collaboration readiness and grant submissions in both groups from baseline through 12-month follow-up, as well as bibliometric data regarding collaboration network size through 21-month follow-up, in both the experimental and control groups. We hypothesized that, in comparison to the TAU control group, participants in the Innovation Lab group, through their immersive experience in collaboratively designing innovative, transdisciplinary research, would experience greater increases in collaboration readiness, transdisciplinarity, grant submissions, and collaboration network size.

## Methods

The study was conducted in accordance with the ethical principles of the Declaration of Helsinki [20] and approved by the institutional review board at the University at Buffalo. All participants provided written informed consent. The study followed the Consolidated Standards of Reporting Trials reporting guideline and was registered on ClinicalTrials.gov (NCT05395286).

### Study design

Participants in each of the two cohorts (2017 and 2018) were randomized to two groups (Innovation Lab vs. TAU control) in a balanced, randomized, parallel-group design. Neither investigators nor participants were blind to treatment group, as this was not feasible.

### Participants

Participants in the RCT were 95 early career scholars (i.e., within 10 years of completing their terminal research degree or residency/fellowship). Eligibility criteria included submitting a complete application and baseline assessment, having a faculty appointment at a CTSA hub institution or regional partner, and selection by the research team for randomization (described below). Participants represented a variety of disciplines and specialties (e.g., anesthesiology, biostatistics, cardiology, communication, emergency medicine, endocrinology, epidemiology, exercise physiology/science, gerontology, informatics, health policy/services, kinesiology, neuroscience, nursing, nutrition, obstetrics and gynecology, oncology, pediatrics, pharmacology, psychiatry, psychology [clinical, developmental, experimental], public health, social work, surgery). Consistent with the focus on early career scholars, most participants (75%) were assistant professors.

### Procedures

#### Scoping survey

To ensure the Innovation Labs focused on topics relevant throughout the CTSA network, we completed a scoping survey. With guidance from NCATS, we surveyed all CTSA hub UL1 and KL2 PIs as well as Collaboration/Engagement, Methods/Processes, and Workforce Development Domain Task Force leads for potential themes. The results of the scoping survey were reviewed by the study team, who made final choices of the topic for each Innovation Lab: “Radical Solutions to the Opioid Misuse Epidemic” for Cohort 1 and “Staying Power: Developing Lifestyle Interventions that Last” for Cohort 2.

#### Recruitment

For each cohort, early career scholars were recruited via email to the leadership of each CTSA hub for distribution, NCATS e-newsletters, announcements on the NCATS-funded Center for Leading Innovation & Collaboration website, Twitter posts, blog posts on EdgeForScholars.org, and circulation of the opportunity twice for each lab topic in the Cutting Edge newsletter which was then distributed to over 40,000 early career scholars, mentors, and academic leaders, to apply to participate in the Innovation Lab via a website (https://www.buffalo.edu/innovationlabs). Women and underrepresented minorities were especially encouraged to apply.

#### Informed consent, application, and baseline assessment

Written informed consent was obtained electronically before the collection of any study data. The application included contact and demographic information and professional details (e.g., degree, field, certification as an early-stage investigator, CTSA hub affiliation) necessary to determine eligibility and characterize the sample. Participants also uploaded their NIH/NSF biosketch and completed six short (150-250 words) essays (e.g., “What do you hope to gain from participating in this Innovation Lab, personally and professionally?” and “What is your personal experience with working in teams? What strengths do you bring to a team effort?”) that were subsequently used to rate and prioritize applicants for randomization (described below).

Following submission of the application, applicants were asked to complete an independent baseline self-report assessment and submit their curriculum vitae and supplemental information (regarding e.g., grant proposals, manuscripts, and publications). Although applicants had to complete the baseline assessment to be considered for randomization, baseline data were maintained independently and not used for selecting applicants for participation in the trial. To minimize response bias on baseline self-report measures, the consent form informed participants of the independence between the application and the baseline assessment.

#### Participant selection meeting

Prior to the selection meeting for each cohort, each application (52 applicants from 26 CTSA hubs in Cohort 1 and 55 applicants from 30 hubs in Cohort 2) was rated by 2-4 raters (an industrial/organizational psychologist, the lab Director, and 1-2 project investigators) on a scale of 1-4 for bringing diverse expertise to the Innovation Lab topic and fit for the Innovation Lab approach (e.g., evidence of tolerance for ambiguity, openness to novelty, and trust in forming new collaborative relationships, factors emphasized in the SciTS literature [21]). During the meeting, facilitated by KnowInnovation, each participant’s application was discussed, and a final consensus determination was made regarding suitability for the Innovation Lab. Applicants with consistently poor scores (∼≤2) were excluded from RCT participation.

#### Stratification and randomization

For each cohort, participants selected for randomization were stratified to balance two groups on disciplinary diversity, degree type (MD, PhD, or other), sex, age, and race and ethnicity. Following stratification, one group was randomly assigned to attend the Innovation Lab; the other group was assigned to the TAU control condition.

#### Intervention: Innovation Labs

The Innovation Labs were facilitated 5-day events (held November 6–10, 2017, in Buffalo, NY, and April 23–27, 2018, in Warrenton, VA) designed to provide experiential learning in the creation of highly novel, transdisciplinary, and transformative collaborative research proposals. Forming collaborative teams, particularly with collaborators outside one’s own discipline who are highly motivated to address the same topic, is an important yet time-consuming and often haphazard process. Innovation Labs are designed to efficiently facilitate this process, bringing together a large, diverse group of scholars interested in the same grand challenge. The Innovation Lab is intended to facilitate the early development of strong teams and proposals within a 1-week time frame, a process that can easily take months of meetings in the typical clinical and translational research environment.

Participants, along with a director (who provided a call to action and scientific leadership), 4-6 subject matter mentors from a range of disciplines (who Socratically catalyzed the creation of new ideas and, at the end of the event, served as a review panel), and KnowInnovation facilitators (who designed the event and managed the process) communally explored the problem space and generated a broad range of ideas. Participants formed transdisciplinary teams to develop and pursue research projects.

**Figure 1.**
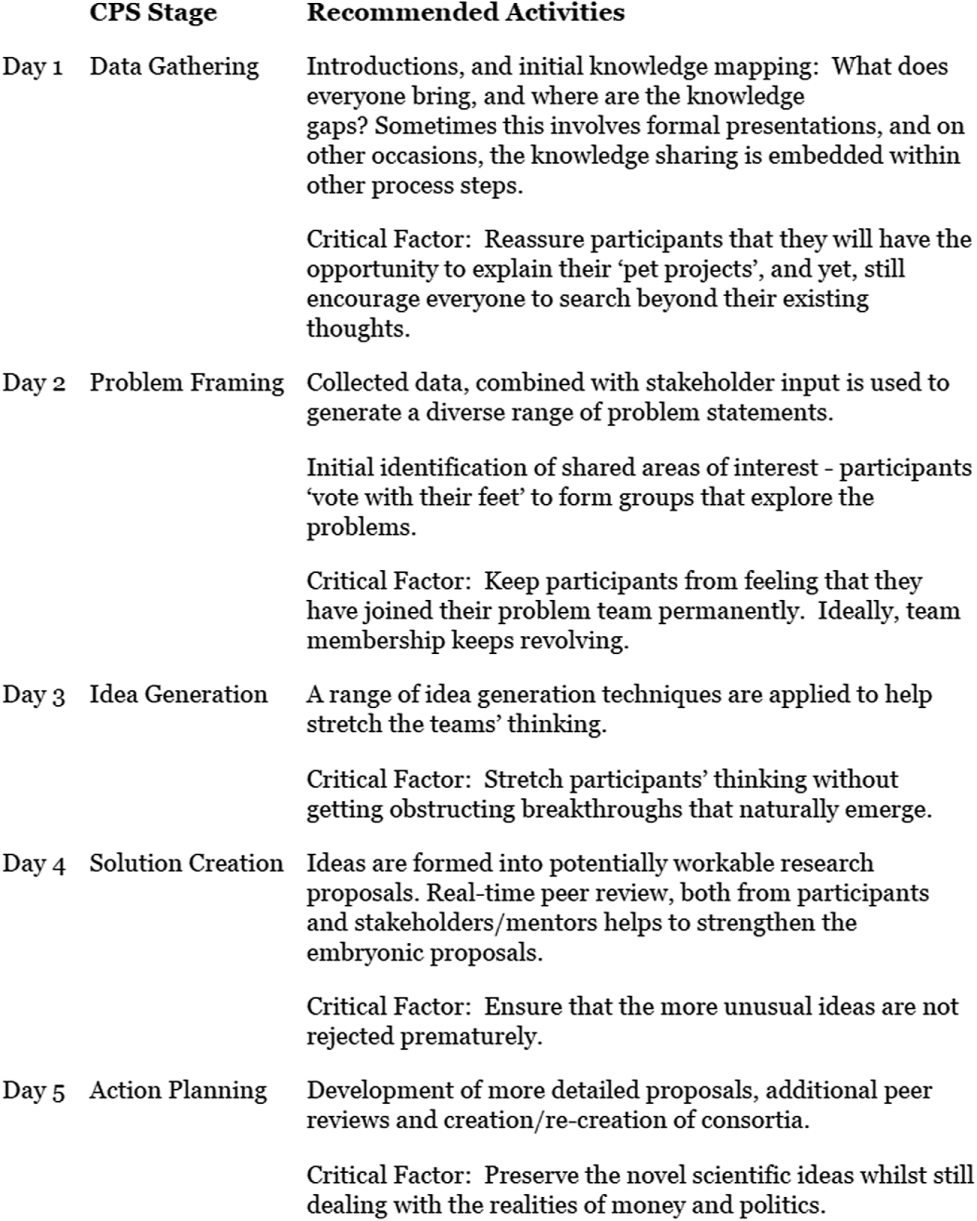
The basic agenda of innovation labs, as driven by a deliberate creative problem-solving process.

The basic structure and process of the Innovation Labs embodied the creative problem-solving process, as shown in Figure 1. Didactic training on collaboration was not provided. Rather, over the course of the week, participants were immersed in an intensive, transdisciplinary collaborative experience. During the first three days of the Innovation Lab, ideas and potential teams evolved rapidly. Participants were repeatedly encouraged to “vote with their feet” as they explored different research ideas with different potential collaborators. This churn of ideas and people was explicitly promoted as a way to reduce premature commitment to particular collaborators or research ideas. The Innovation Lab also provided numerous informal opportunities to socialize, including 3 communal meals per day, outings (e.g., to a museum), and recreational activities (e.g., a soccer friendly), providing participants additional opportunities to determine with whom they are intellectually and interpersonally compatible.

On Day 1, participants were guided to introduce themselves and their expertise and to understand the expertise and perspectives of others (e.g., a 1:1 getting-to-know-you activity, followed by an introduction of the other member of the dyad to the larger group). Participants communally discussed the Director’s call to action and developed a preliminary set of interesting questions, relevant data points, and proposed solutions and challenges, thereby developing a shared understanding of the problem space and knowledge in the room. This process, interspersed with 1-3 provocateur presentations (to encourage participants to think more broadly about the problem space), continued on Day 2.

On Day 3, multiple rounds of candidate projects were presented to the entire Lab as 1-page posters, and participants were encouraged to form preliminary teams (some participants join multiple teams). After the presentations, each participant was encouraged to choose a primary and secondary project to explore in subsequent discussion. Following this discussion, preliminary teams announce the project on which they will be working (a few participants worked on two teams/projects).

Beginning on Day 4 and continuing into Day 5, teams focused on developing a specific research proposal, with three iterative rounds of group working time, followed by presentation and feedback from other participants and Lab mentors. On Day 4, teams typically worked together late into the evening as they refined their proposals, with the opportunity to discuss the project in 30-minute mentor clinics. Changes to team membership, while less common at this point, continued to occur as warranted by project needs and participant preferences. On Day 5 (which ended by 2 p.m.), teams completed a final round of presentations, with feedback from other participants and mentors, after which the director offered closing remarks.

During the week of the Innovation Lab, participants in the Lab group (but not the control group) were asked to provide daily feedback via REDCap.

To facilitate the continued development of research proposals by Innovation Lab teams, we provided an opportunity to apply for pilot funds (up to $3,000 for the 2017 Lab, $4,000 for the 2018 Lab) to the collection of preliminary data and/or team meetings.

#### Follow-up assessments

For each cohort, follow-up assessments were completed via REDCap at the conclusion of the Innovation Lab (end of treatment; EOT) and 6 and 12 months later (6-month and 12-month follow-up). Participants were provided with modest remuneration ($50US) for completing each of these three assessments.

### Outcome measures

Table 1 summarizes the assessment frame for primary and secondary outcome measures.

**Table 1.** Assessment details for primary and secondary outcome measures

Measure	Domain	Self-report or objective	Groups Assessed	Assessment Points/Windows
**Primary Outcomes**				
MATRICx motivators	Collaboration readiness	Self-report	Both	Baseline, EOT, 6 M, 12 M
MATRICx barriers	Collaboration readiness	Self-report	Both	Baseline, EOT, 6 M, 12 M
Transdisciplinary orientation (TOS)				
Coauthor network size	Collaboration behavior	Objective (bibliometric)	Both	Pre (−21 – −4 months before EOT) and post (4–21 months after EOT)
**Secondary Outcomes**				
IL Team/proposal formation	Process measure	Self-report	IL only	EOT
IL Pilot fund request	Process measure	Objective	IL only	∼ 6 M
IL feedback	Intervention feedback	Self-report	IL only	EOT, 6 M, 12 M
Collaboration self-efficacy	Collaboration readiness	Self-report	Both	Baseline, EOT, 6 M, 12 M
Grant submissions	Collaboration behavior	Self-report	Both	Baseline, EOT, 6 M, 12 M

6 M = 6-month follow-up; 12 M = 12-month follow-up; EOT = end of treatment; IL = Innovation Lab; MATRICx=Motivation Assessment for Team Readiness, Integration, and Collaboration; TOS = Transdisciplinary Orientation (TDO) Scale.

Collaboration self-efficacy was added after Cohort 1 completed EOT; thus, only the 2018 cohort data are presented, and the measure is considered secondary. Team/proposal formation and pilot fund request were not pre-registered, but they have been added to address comments from an anonymous reviewer.

#### *Innovation Lab feedback* (secondary outcomes; self-report)

Innovation Lab participants were asked to provide feedback each day of the Lab and again at 6- and 12-month follow-up. Key secondary outcomes were EOT (Friday of the Innovation Lab) and 6- and 12-month ratings of the degree to which “The Lab met the goal of forming new transdisciplinary collaborations,” “The Lab met the goal of developing novel grant proposals,” “I would recommend an Innovation Lab to a colleague,” and “My experience in the Innovation Lab will have / is having a positive impact on my work” (0 = Strongly Disagree to 5 = Strongly Agree).

#### *Collaboration readiness* (primary outcomes; self-report)

At baseline, EOT, and 6- and 12-month follow-up, participants were asked to complete measures of collaboration readiness.

The Motivation Assessment for Team Readiness, Integration, and Collaboration (MATRICx [22]) assesses 17 perceived benefits/motivators (e.g., “Collaboration enables scholarly problems to be solved more quickly”) and 31 barriers (e.g., “I lose independency by collaborating”) on a 4-point scale. Mean scores were computed as the mean of the items on each scale. Internal consistency (Cronbach’s *α*) was 0.89 and 0.88 for the benefits and barriers scales at baseline, and the two scales only were modestly negatively correlated (*r* = −.35).

The Transdisciplinary Orientation (TDO) Scale [23] is a 12-item scale, with two subscales, values, attitudes, and beliefs (e.g., “…openness to diverse disciplinary perspectives…;” *α* = 0.84 at baseline) and conceptual skills and behaviors (e.g., “…ability to create conceptual frameworks that bridge multiple fields;” *α* = 0.89 at baseline). Items are rated on a five-point scale (1=“Strongly Disagree,” 5=“Strongly Agree”). Given the high correlation between the subscales (*r* = 0.80 at baseline), a total TDO score was computed across all items.

A measure of collaboration self-efficacy was added to the protocol after Cohort 1 completed EOT; thus, only the 2018 cohort data are presented for this secondary outcome. The 8-item measure (*α* = 0.86 at baseline) was based on Spring *et al*.’s teamscience.net [24] measure (first 6 items) and personal communication with Kevin Wooten (last 2 items), assesses confidence (1–10) in the ability to perform collaboration-related tasks (e.g., “… assemble and manage a cross-disciplinary research team,” “…work with colleagues to develop a strong collaboration plan…”).

#### *Collaboration network size* (primary outcome; objective)

Collaboration network size was operationalized as the number of unique coauthors in PubMed during 18-month pre- and post-treatment periods (after excluding articles published within ±3 months of the Innovation Lab). Author lists for each article in the pre- and post-treatment periods were downloaded and reconciled. The PubMed legacy interface we employed limited the number of author names in the downloaded citation to 25; however, this does not seem problematic, as a spot check of articles suggested that very few had more than 25 authors.

#### *Grant submissions* (secondary outcome; self-report)

Participants indicated the number of grants submitted (0, 1, 2, 3, 4, 5+) in the past six months at baseline and 6- and 12-month follow-up; a 3-month interval was used at the EOT assessment to avoid overlap with the baseline period. Though participants were asked to provide additional details (including the names and affiliations of collaborators), many participants chose not to complete these more burdensome components. Therefore, the analysis focused on the number of grants submitted. Participants (*n* = 40) who did not report the number of grants submitted were coded as zero for the ITT analysis. For the post-treatment period, the number of grants submitted at 6- and 12-month follow-up were summed. To make the pre-treatment period (which covered only 9 months) comparable to the post-treatment period, the number of grants at baseline and EOT were summed, divided by 9, and then multiplied by 12.

### Statistical analysis

Analysis of measures of collaboration readiness included Group × Time (baseline, EOT) analyses of variance (ANOVAs) to assess the immediate effect of Innovation Lab participation. To evaluate change from EOT to 6- and 12-month follow-up, we conducted multi-level growth models, with time as a Level 1 predictor (linear and quadratic trends were evaluated; quadratic was retained in the final model only when it accounted or incremental variance beyond the linear contrast) and Group and the Group × Time interactions as Level 2 predictors.

Collaboration network size and number of grant submissions were analyzed in 2 Group × 2 Time (pre, post) ANOVAs. All participants had complete data for collaboration network size. For the number of grant submissions, the primary analysis was conducted on the ITT data (all participants; missing = 0); a supplemental analysis evaluated only participants with complete data.

## Results

### Baseline characteristics

Participants were, on average, 38 years old. The majority self-reported being female (74%) and white (82%). The Innovation Lab and TAU Control groups were comparable on all baseline characteristics (Table 2).

**Table 2.** Participant characteristics in all cohort x group conditions, as well as overall

	Innovation Lab			TAU Control			Grand Total
	Cohort 1^abc^	Cohort 2^bd^	Total	Cohort 1	Cohort 2^e^	Total	
N randomized	24	23	47	24	24	48	95
Sex, % female	73.9%	69.6%	71.7%	75.0%	78.3%	76.6%	74.2%
**Race**							
African/AA	0.0%	4.5%	2.2%	0.0%	4.3%	2.1%	2.2%
Asian	8.7%	13.6%	11.1%	12.5%	13.0%	12.8%	12.0%
White	82.6%	77.3%	80.0%	87.5%	78.3%	83.0%	81.5%
Multiple/Other	8.7%	4.5%	6.7%	0.0%	4.3%	2.1%	4.3%
Ethnicity, Hispanic	0.0%	13.6%	6.5%	0.0%	13.0%	6.4%	6.5%
Age, mean (SD)	37.7 (4.8)	38.9 (6.3)	38.3 (5.6)	37.1 (5.8)	38.3 (6.7)	37.7 (6.2)	38.0 (5.9)
**Terminal degree**							
MD	33.3%	13.0%	23.4%	29.2%	13.0%	21.3%	22.3%
PhD	58.3%	82.6%	70.2%	54.2%	82.6%	68.1%	69.1%
MD-PhD	4.2%	4.3%	4.3%	12.5%	4.3%	8.5%	6.4%
Other	4.2%	0.0%	2.1%	4.2%	0.0%	2.1%	2.1%

a One participant did not disclose their sex.

b One participant did not disclose their race.

c One participant did not disclose their age.

d One participant did not disclose their ethnicity.

e Though 24 ppts were randomized to control, one ppt withdrew at end of treatment and asked that all their data be deleted. Therefore, the table provides information based on the remaining 23 participants.

### Retention

**Figure 2.**
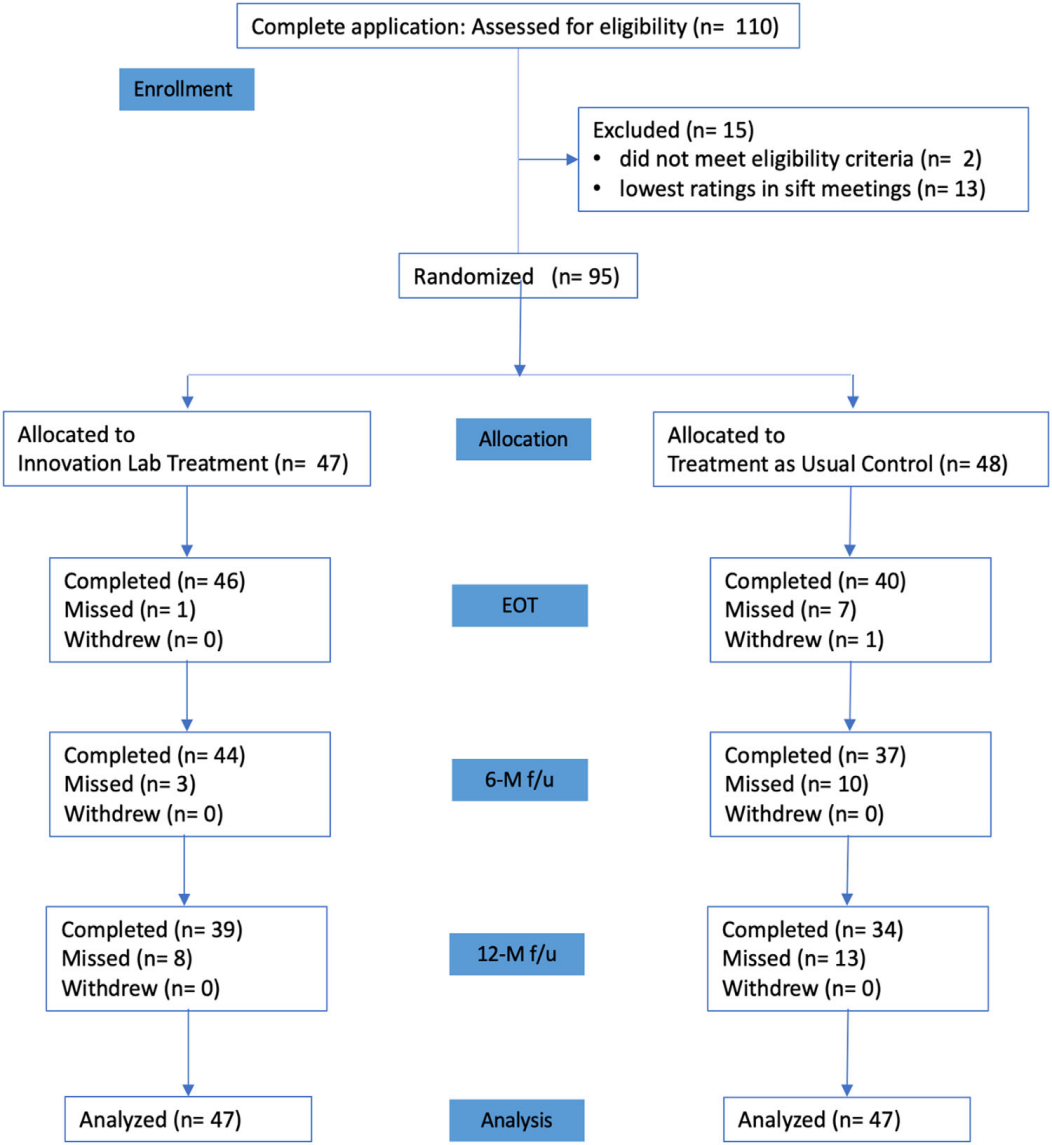
Flow diagram of trial recruitment and eligibility evaluation, intervention randomization, follow-up, and analysis.

Retention was significantly lower in the TAU Control Group compared to the Innovation Lab Group at EOT (40/48 [83%] and 46/47 [98%]) and 6 M (37/48 [77%] and 44/47 [94%]), but not at 12 M (34/48 [71%] and 39/47 [83%]) (see Fig. 2) [*χ* ^2^ [1] = 5.9, 5.2, and 2.0, *ps* = 0.02, 0.02, 0.16, respectively].

### Preliminary, uncontrolled Innovation Lab outcomes

#### Formation of new collaborative teams/proposals

Over 90% of participants randomly assigned to the Innovation Labs group received the intervention (i.e., 22/24 in 2017 and 21/23 in 2018 attended the 5-day events). Descriptively, the Innovation Labs led to the formation of 7 collaborative teams/proposals in Cohort 1 and 5 collaborative teams/proposals in Cohort 2. Thus, the opportunity for immersive experiential learning regarding collaboration formation and elaboration was realized for most Innovation Lab participants. Seven of the 12 teams applied for and received pilot funding from us to further their collaborative proposals.

#### Innovation Lab feedback

At EOT, the majority of Innovation Lab participants Agreed or Strongly Agreed that: (a) the Lab met the goal of forming new transdisciplinary collaborations (77% [27/35]), (b) the Lab met the goal of developing novel grant proposals (63% [22/35]), (c) they would recommend an Innovation Lab to a colleague (85% [29/34]), and (d) the Innovation Lab experience will have a positive impact on their work at their home institution (86% [30/35]); No more than 14% of Innovation Lab participants disagreed with any of these statements at EOT.

Feedback about attending the Innovation Labs became less positive across the follow-up period (Table 3) [time linear (EOT vs. 12 M) *Fs* (1,65.2–68.2) = 45.3, 16.6, 11.3, and 13.8, all *ps* < .001]. At 12 M, average ratings for forming new collaborations and developing novel grant proposals were, on average, just below Mildly Agree, and average ratings for recommending an Innovation Lab and positive impact of the Innovation Lab were just below Agree.

**Table 3.** Feedback from Innovation Lab participants at end of treatment (EOT), 6-month (6 M) follow-up, and 12-month (12 M) follow-up. Values are mean (standard deviation)

Feedback prompt	EOT	6 M	12 M
The Lab met the goal of forming new transdisciplinary collaborations	4.3 (0.8)	3.7 (1.0)	2.9 (1.4)
The Lab met goal of developing novel grant proposals	3.7 (1.1)	3.4 (1.1)	2.6 (1.4)
I would recommend an Innovation Lab to a colleague	4.4 (0.9)	4.3 (0.9)	3.8 (1.4)
My experience in the Innovation Lab will have (is having) a positive impact on my work	4.4 (0.8)	4.1 (0.9)	3.7 (1.4)

6 M = 6-month follow-up; 12 M = 12-month follow-up; EOT = end of treatment. *N* = 34-35 at EOT, 35-36 at 6 M, and 33-34 at 12 M (response rate of 70%–77%).

Response range is 0 (strongly disagree) to 5 (strongly agree).

### Collaboration readiness

**Figure 3.**
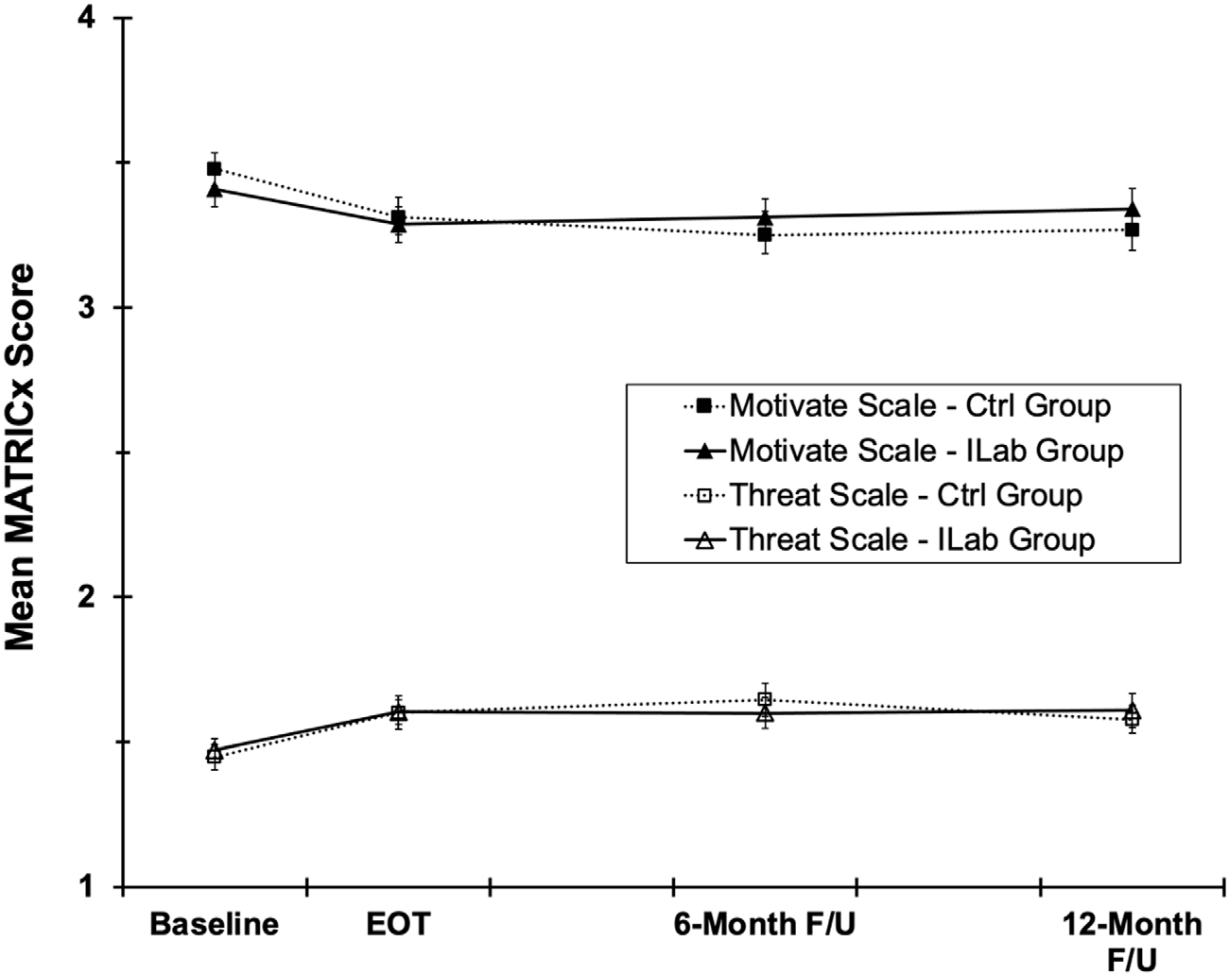
Mean MATRICx perceived collaboration motivators/benefits and threats/barriers scores for all group x time conditions. Error bars are ± 1 standard error.

Perceived motivators/benefits of collaboration on the MATRICx were near the top of the scale at baseline and declined modestly at EOT (Fig. 3) [*F*(1,83) = 8.3, *p* = .005]. However, this decline did not significantly vary between groups [Group and Group × Time *Fs* < 1]. On average, perceived motivators/benefits of collaboration remained stable from EOT through 12 M follow-up [time, group, and Group × Time *Fs* < 2.3, *ps* > .13].

Perceived collaboration threats/barriers on the MATRICx were near the bottom of the scale at baseline and increased modestly from baseline to EOT [*F*(1,83) = 60.2, *p* < .001]. However, this increase did not significantly vary between groups [Group and Group × Time *Fs* < 1]. On average, perceived collaboration threats/barriers did not significantly change from EOT through 12 M follow-up [time, group, and Group × Time *Fs* < 1, *ps* > .30].

**Figure 4.**
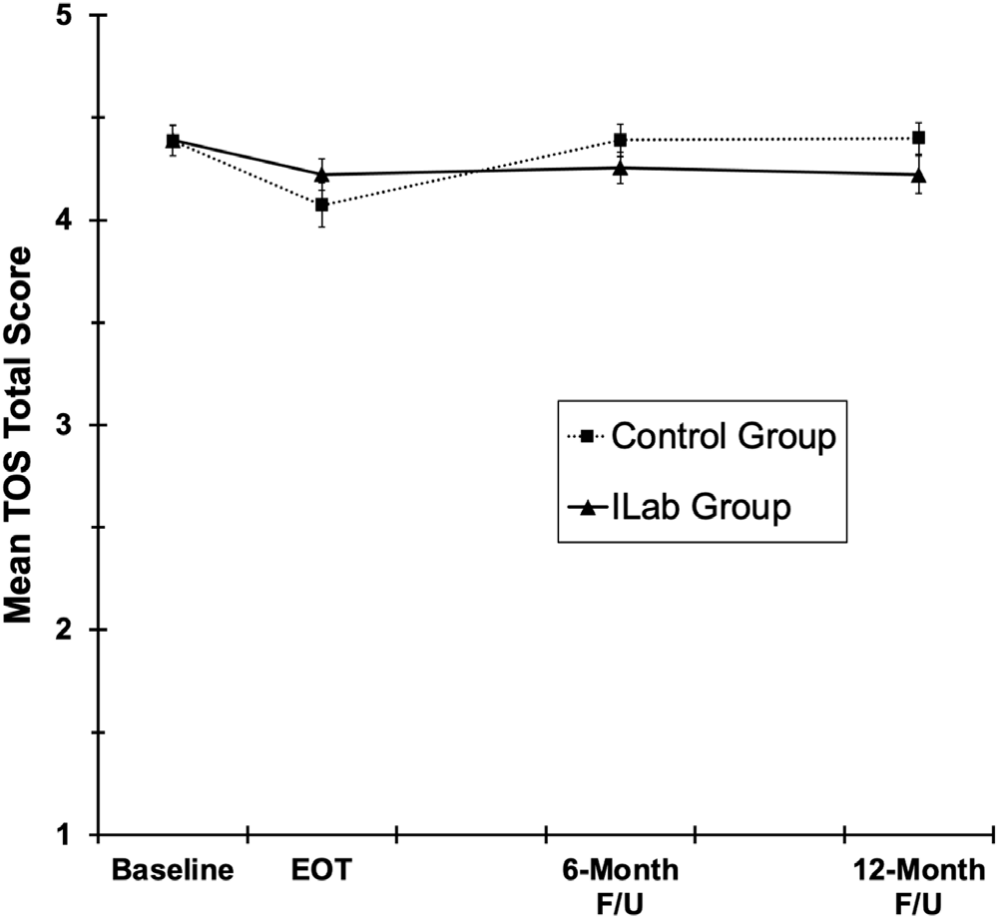
Mean TOS transdisciplinary orientation total score for all group x time conditions. Error bars are ± 1 standard error.

As for the MATRICx motivators/benefits, mean transdisciplinary orientation on the TOS was near the top of the scale at baseline and modestly declined from baseline to EOT (see Fig. 4), but the two treatment groups did not differ overall or in the magnitude of the decline [time, group, and Time × Group *Fs*(1,84) = 17.7, 0.7, and 1.3, *ps* < 0.001, 0.41, and 0.26, respectively]. During the post-treatment period (EOT through 12 M follow-up), TOS scores tended to rebound towards baseline levels in the TAU Control group but not in the Innovation Lab Group, resulting in significant Group × Time Linear interaction [*F*(1,82.7) = 4.6, *p* = .03]. However, the two groups did not significantly differ in transdisciplinary orientation at EOT, 6 M, or 12 M [*ps* = 0.14, 0.77, and 0.13, respectively].

**Figure 5.**
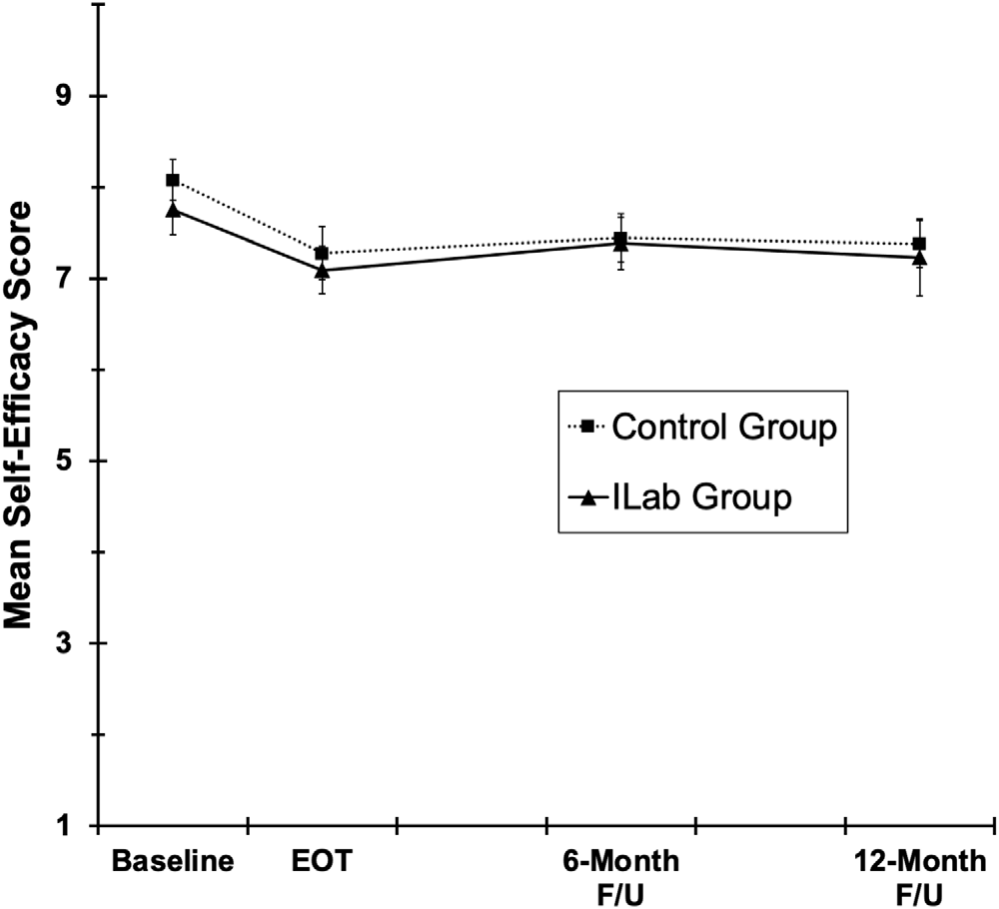
Mean collaboration self-efficacy scores for all group x time conditions in the 2018 cohort. Error bars are ± 1 standard error.

Average self-reported collaboration self-efficacy was ∼ 8/10 at baseline and declined on average about 0.7 points from baseline to EOT, a decline that was comparable for the Innovation Lab and TAU Control Groups (Fig. 5) [time, group, and Time × Group *Fs*(1,38) = 11.0, 0.7, and 0.2, *ps* < 0.002, 0.43, and 0.70, respectively]. From EOT through 12 M follow-up, collaboration self-efficacy remained relatively consistent across time and groups [time, group, and Time × Group *Fs* < 0.2, *ps* > .67].

### Collaboration network size

**Figure 6.**
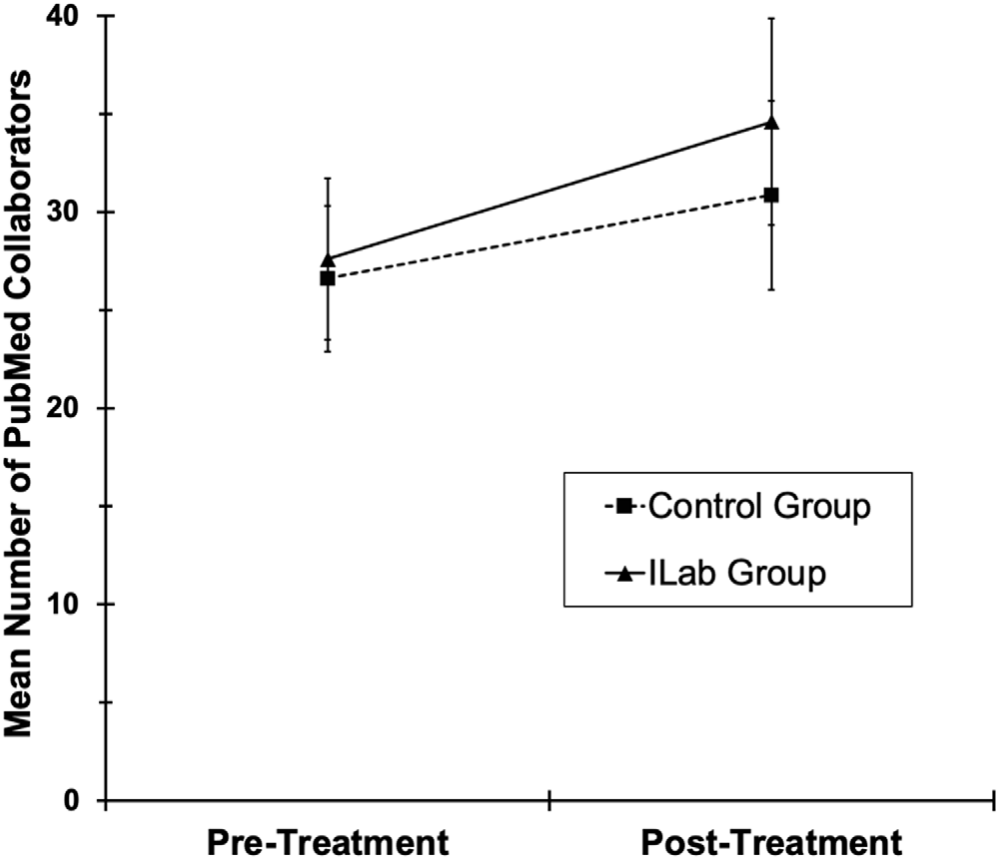
Mean number of coauthors from PubMed for both treatment groups during pre-treatment (18 months, spanning from EOT minus 21 months through EOT minus 4 months) and post-treatment (18 months, spanning from EOT plus 4 months through EOT plus 21 months). Error bars are ± 1 standard error.

On average, the number of publication coauthors associated with each participant in PubMed increased from the 18-month pre-treatment period to the 18-month post-treatment period (Fig. 6) [*F*(1,92) = 11.0, *p* < .001]. However, this growth did not significantly vary as a function of treatment group [group and Group × Time *Fs* < 1].

We conducted two post hoc exploratory analyses. First, given the possibility that group differences in coauthor networks might take longer to emerge, we conducted simulations in which the growth in the collaborator network doubled at a subsequent assessment. Second, given the marked within-group heterogeneity in the number of coauthors within each group (note the error bars in Fig. 6), we reduced the maximum number of collaborators per article from 25 to 10; this resulted in a more normal distribution of the collaboration network size. Nevertheless, as in the primary analysis, there was no evidence of significant group differences in collaboration network size in either of these post hoc exploratory analyses.

### Grant submissions

**Figure 7.**
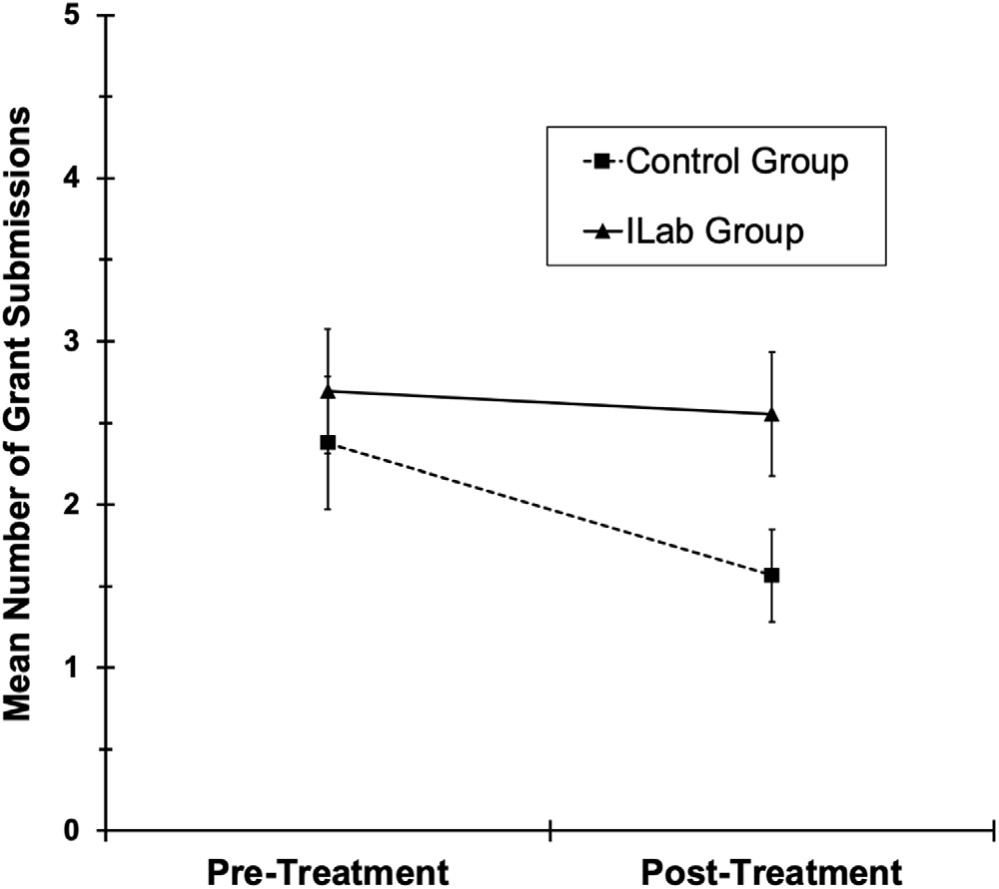
Mean number of self-reported grant submissions (ITT data; missing = 0) for both treatment groups during the year preceding (pre-treatment) and following (post-treatment) the Innovation Lab. Error bars are ± 1 standard error.

In the ITT analysis of grant submissions, the general decline in submissions from the pre-treatment year to the post-treatment year tended to be driven by the TAU control group (see Fig. 7) [time *F*(1,92) = 5.3, *p* = .02; Group × Time *F*(1,92) = 3.3, *p* = .07]. The Innovation Lab and TAU Control Groups did not significantly differ at pre-treatment [*p* = .88], but grant submissions during post-treatment were higher in the Innovation Lab Group compared to the Control Group [*p* = .04].

In the analysis of only participants with complete data for grant submissions at all assessment waves (*n* = 25 Control and 29 Innovation Lab participants), the same general pattern of means was observed, but the Group × Time interaction was not significant [*F*(1,52) = 1.8, *p* = .18], and the groups did not significantly differ at either pre- (means[SEs] = 2.8 [.57] and 3.2 [.62] for Innovation Lab and Control) or post-treatment (means = 3.4 [.45] and 2.7 [.48] for Innovation Lab and Control) [*ps* = 0.60 and .28].

## Discussion

We sought to determine the ability of 5-day, immersive Innovation Labs to produce an increase in short- to intermediate-term enhancement of collaboration among early career faculty scholars in the CTSA network. The standard of evaluation is a critical factor in determining the success of the approach. When focusing on the short-term, uncontrolled outcomes typical of the field at present, the Innovation Labs were clearly successful: 12 new cross-disciplinary teams were formed, 7 of the 12 applied for and received pilot funding from us, and short-term evaluations of the Innovation Labs were quite positive, with most participants agreeing or strongly agreeing that the Lab met the goal of forming new transdisciplinary collaborations and that the experience will have a positive impact on participants’ work. However, these preliminary outcomes are weak in that they were assessed only in the Innovation Labs group, precluding comparisons to the control group randomized to “TAU.” Indeed, measures that did allow for direct comparisons between groups revealed modest evidence of a beneficial impact of Innovation Labs for early career scholars. Specifically, the number of self-reported grants (a secondary outcome measure) was larger in the Innovation Lab group compared to the control group; however, this difference was observed only in the ITT analysis, which may have been biased against the control group, for which attrition was greater. Most importantly, the groups did not differ at follow-up on the primary outcome measures, subjective measures of collaboration readiness, and the objective size of participants’ collaboration networks. Below, we consider the implications of the findings of the present study, with an emphasis on lessons learned from this initial collaboration RCT.

### Innovation Labs and collaboration readiness

In an early career scholar sample, which has limited collaboration experience compared to more established investigators, we hypothesized that Innovation Labs, which facilitate trust and team-building and highlight the potential of cross-disciplinary collaboration, would enhance collaboration readiness. However, across multiple measures (MATRICx, TOS, and collaboration self-efficacy) and over time, the Innovation Labs and TAU control groups did not differ in collaboration readiness. The interpretation of these findings is complicated by restricted range on all measures of collaboration readiness. That is, participants in both groups tended to be near the ceiling on perceived motivators/benefits of collaboration, transdisciplinary orientation, and collaboration self-efficacy and near the floor on perceived barriers/threats to collaboration.

Relatively extreme scores could be due to response bias – that participants believed they would only be selected for the project if they endorsed high levels of collaboration readiness. Indeed, despite the explicit separation of collaboration readiness outcomes from the application materials, scores on all collaboration readiness measures drifted modestly closer to the middle of each scale from baseline to EOT. However, scores stabilized or even moved slightly more extreme over the one-year follow-up period. Therefore, it seems more plausible that participants truly perceived themselves as “collaboration ready.” It is certainly possible that only people who perceived themselves to be high in collaboration readiness and were motivated to participate in cross-disciplinary collaborations applied to the Innovation Labs study. Consistent with this interpretation, transdisciplinary orientation scores were nominally higher in the present sample (at all time points) than in the original TOS development and validation samples (means = 4.09 and 3.94 [23]), despite the present sample being younger by a decade, on average (38 vs 49 years).

Regardless of the reason for the extreme scores, they left little room for demonstrating enhancement of collaboration readiness by the Innovation Labs. Future research on the impact of team science interventions on collaboration readiness may require refinement of the field’s measures to be more sensitive and discriminating at the upper and lower ends of the scales. More generally, the present data call for further work to evaluate (and perhaps improve) the ability of collaboration readiness measures to prospectively predict individual differences in collaboration behavior. Interestingly, team science interventions may best foster collaboration readiness among scholars with low-to-moderate baseline levels of collaboration readiness, recognizing that it may be more difficult to recruit such people to collaboration-focused trials.

### Team formation and maintenance/products

Initial formation of new collaborations was evident in each of the Innovation Labs (see also, e.g., [11]). Though we did not collect data on the formation of new collaborations among the TAU control group during the two Innovation Lab weeks, it seems likely that the intervention directly caused the formation of new teams during the 1-week treatment.

However, there was substantially less evidence for the impact of Innovation Labs on collaboration readiness and behavior more generally, including the impact on objective (bibliometric) collaboration network size. Though it is possible that the study was under-powered to detect true differences in collaboration networks, as the study was only powered to detect medium effect sizes, the group difference in network size was not even close to statistical significance. The most parsimonious interpretation of these null findings is that the Innovation Labs were not efficacious in maintaining nascent cross-disciplinary, early career collaborations formed in the Labs and stimulating new collaborations through the development of grant proposals and published manuscripts. This hypothesis is supported by the finding that Innovation Lab participants evaluated the Labs less favorably over the one-year follow-up. Additional maintenance components, such as comprehensive collaboration plans [5] to address the many challenges faced by cross-disciplinary teams, may be warranted. Alternatively, given the multi-year lag in productivity that can occur when forming new cross-disciplinary collaborations [25], grant submissions and patterns of coauthorship may be too distal from the intervention to serve as reasonable targets for early-stage evaluation of team science interventions. This may be particularly true for early career scholars who are diligently working to develop their independent research careers.

Looking ahead, the field may benefit from considering stage models for behavioral treatment development, with an emphasis on identifying a set of common, short-term efficacy signals that can be used to evaluate and refine promising interventions [26], including outcomes that can be passively collected [27]. For example, with this perspective in mind, we added a measure of self-reported collaboration self-efficacy to the assessment protocol after our trial began. The fact that collaboration self-efficacy was not enhanced by the Innovation Lab leads us to believe that the standard Innovation Lab experience provided in this RCT may not have been optimized for enhancing participants’ understanding, development, and implementation of collaboration principles, competencies, and processes (e.g., [28]). That is, participants may have been so focused on developing a collaborative proposal within the 5-day window that there was limited opportunity to recognize and reflect on the underlying processes and integrate them into their approach to collaboration after the lab ended. In the time since we conducted the RCT, KnowInnovation has modified the training/intervention version of Innovation Labs to provide daily opportunities to reflect on and discuss how to use creative problem-solving in future collaborations. Whether these modifications had the intended effect has not been formally evaluated – but a series of such iterative evaluations and refinements will likely be needed to produce collaboration interventions with lasting impact. Even if a refined Innovation Labs approach proved efficacious, its’ high-intensity (5 contiguous days), expensive (cost of facilitators, travel, room, and board) nature might limit its cost-effectiveness and feasibility for implementation in the absence of further refinements, such as virtual components and/or training additional, lower-cost facilitators.

More generally, a major investment from NCATS and the CTSA network is long overdue for the science of team science. Rigorous controlled evaluation of a range of promising team science interventions is necessary to formally examine and compare their efficacy, reach, and cost-effectiveness. Unfortunately, at present there is little funding available for such programmatic team science intervention development research.

### Additional lessons for conducting clinical trials with scholar participants

A final set of lessons learned concerns generalizability and the challenges of recruiting and retaining early career scholars in longitudinal SciTS intervention research. As the 2015 National Academies monograph on *Enhancing the Effectiveness of Team Science* notes, rigorous experimental research requires “access to practicing scientists” (p. 12) [4]. Our experience with the present trial suggests diverting scholars from existing demands may create a real bottleneck. Far fewer scholars applied to the current Innovation Labs than to past Labs [11–17], despite extensive outreach via social media and newsletters, as well as email to leadership at each CTSA hub. Although many factors may have contributed to this difference, we hypothesize (in part based on anecdotal feedback) that beginning the application with a consent form that clearly described that highly qualified applicants would be randomized served as a disincentive. From an ethical perspective, informed consent is essential. Yet, given that more than half of the applicants stopped the application process at the consent stage, it also seems essential to better understand scholars’ decisions about participation in RCTs in order to enhance participation rates in future trials.

Limitations of the generalizability of this randomized trial may inform future work. As in most randomized trials, participants were not representative of the broader population. In addition to self-selection of individuals motivated to engage in collaborative research, the topic of any given Innovation Lab is more relevant to some scholars and disciplines than others. Participants were also predominantly female and white, limiting generalizability to males and people of color. In future studies, additional efforts may be needed to reach participants with these demographic characteristics. Finally, while the average age of 38 years may seem unusual, it likely is not, given the increasingly protracted training period for biomedical and behavioral sciences researchers [29].

Even though nominal retention rates were relatively strong, retention was lower in the control group than the intervention group at EOT and 6-month follow-up. This issue is not uncommon in RCTs, but because RCTs are not yet common in SciTS research, it is important to address for internal and external validity of the work to be optimal. Qualitatively, obtaining reasonable follow-up rates required considerable effort, relative to our non-SciTS RCT experience. In addition to the remuneration of $50 per assessment for EOT, 6- and 12-month follow-up, we: (a) sent “save the date” emails about upcoming assessments that reminded participants of the importance of strong follow-up rates for interpreting the data, (b) employed repeated email reminders to encourage completion of assessments that included information about completion rates to date and, as the project matured, notices regarding streamlining of assessments to reduce participant burden (e.g., requesting less detail on recent scholarly products), and (c) later in the project, followed up with personal email and phone calls from the PI (with IRB approval) to participants who had not completed an assessment by the target date.

Our experience suggests that it would be helpful to conduct scientific studies of the barriers and facilitators of scholar *participation* and retention in clinical trials. Based on the more typical Innovation Lab approach [11–17], one method of enhancing both initial participation and retention in both treatment and control groups may be to have a funding announcement that is open only to participants who complete the study. Although we provided an opportunity for Innovation Lab participants to apply for modest ($3,000-$4,000) pilot funds, a larger pilot fund competition open to both treatment groups may have increased retention and could have the added benefit that proposal submission rate and formal evaluation of transdisciplinarity and novelty could serve as the type of short-term efficacy signals called for above. In the absence of such opportunities for greater short-term return on participants’ investment, scholars may require ample remuneration to participate in team science RCTs, particularly those that require substantial time commitment.

### Summary and conclusions

Our study is notable as the first RCT of any collaboration intervention targeting clinical and translational scientists. Innovation Labs rapidly (1 week) led to the initial formation of cross-disciplinary teams of early career scholars and were well received by participants. In addition, a beneficial impact of Innovation Labs over the control condition was observed in the self-reported number of grant proposals in the intent-to-treat sample. However, the impact on collaboration readiness and collaboration network size (our primary outcomes) was not evident in this cohort of early career investigators with a high level of collaboration readiness at the outset. Based on these results, we hypothesize that including components that foster collaboration maintenance (e.g., substantial dedicated funding opportunities) may be beneficial in enhancing cross-disciplinary collaboration among geographically dispersed teams of early career scholars. More generally, as a preliminary randomized, controlled evaluation in the SciTS field, the present study serves as a guide to future research on CTR team science interventions. Indeed, given the importance of fostering effective collaborations for advancing clinical and translational research, it is critical that NCATS and other funding agencies invest more heavily in evaluating which of the many promising collaboration tools and interventions available are efficacious – and which are not.
